# Inflammatory Signaling Pathways in Preleukemic and Leukemic Stem Cells

**DOI:** 10.3389/fonc.2017.00265

**Published:** 2017-11-13

**Authors:** Shayda Hemmati, Tamanna Haque, Kira Gritsman

**Affiliations:** ^1^Department of Medicine, Albert Einstein College of Medicine, Bronx, NY, United States; ^2^Department of Cell Biology, Albert Einstein College of Medicine, Bronx, NY, United States; ^3^Department of Oncology, Montefiore Medical Center, Bronx, NY, United States

**Keywords:** inflammatory, preleukemic, leukemic stem cell, toll-like receptor, tumor necrosis factor, interferon, interleukin, NF-κB

## Abstract

Hematopoietic stem cells (HSCs) are a rare subset of bone marrow cells that usually exist in a quiescent state, only entering the cell cycle to replenish the blood compartment, thereby limiting the potential for errors in replication. Inflammatory signals that are released in response to environmental stressors, such as infection, trigger active cycling of HSCs. These inflammatory signals can also directly induce HSCs to release cytokines into the bone marrow environment, promoting myeloid differentiation. After stress myelopoiesis is triggered, HSCs require intracellular signaling programs to deactivate this response and return to steady state. Prolonged or excessive exposure to inflammatory cytokines, such as in prolonged infection or in chronic rheumatologic conditions, can lead to continued HSC cycling and eventual HSC loss. This promotes bone marrow failure, and can precipitate preleukemic states or leukemia through the acquisition of genetic and epigenetic changes in HSCs. This can occur through the initiation of clonal hematopoiesis, followed by the emergence preleukemic stem cells (pre-LSCs). In this review, we describe the roles of multiple inflammatory signaling pathways in the generation of pre-LSCs and in progression to myelodysplastic syndrome (MDS), myeloproliferative neoplasms, and acute myeloid leukemia (AML). In AML, activation of some inflammatory signaling pathways can promote the cycling and differentiation of LSCs, and this can be exploited therapeutically. We also discuss the therapeutic potential of modulating inflammatory signaling for the treatment of myeloid malignancies.

## Introduction

Several known preleukemic disorders, including myelodysplastic syndrome (MDS) and the myeloproliferative neoplasms (MPNs), are characterized by acquired cytogenetic abnormalities or molecular alterations in hematopoietic stem cells (HSCs) (1, 2). These alterations result in altered or ineffective hematopoiesis, and varying degrees of bone marrow fibrosis, ultimately leading to morbidity and decreased life expectancy. Preleukemic stem cells (pre-LSCs) have a selective growth advantage over normal HSCs, but are still capable of normal differentiation [reviewed in Ref. (3)]. In acute leukemias, LSCs have the distinct property to undergo self-renewal, but these cells can only differentiate into leukemic blasts [reviewed in Ref. (4)]. Both pre-LSCs and LSCs have been implicated in posttreatment relapse in leukemia patients. Abnormalities in inflammatory signaling have been noted in both preleukemic conditions (MDS/MPN) and in acute myeloid leukemia (AML), suggesting an important role for inflammatory signaling in these conditions. Inflammatory signaling can occur both in hematopoietic cells and in the hematopoietic niche, significantly altering the crosstalk between hematopoietic cells and their microenvironment [reviewed in Ref. (5)]. Interestingly, some of the same inflammatory pathways may actually promote the differentiation and loss of self-renewal of LSCs in AML.

## Clonal Hematopoiesis, Mutations in Epigenetic Modifiers, and Inflammation

Clonal hematopoeisis of indeterminate potential (CHIP) is a recently characterized entity, which describes the increased rate in the acquisition of somatic mutations in hematopoietic cells with increasing age, in the absence of cytopenias or morphologic bone marrow fibrosis or dysfunction (6). While many of these mutations are commonly seen in MDS or AML, and their presence in hematopoietic cells is associated with an increased risk of developing hematologic malignancies, the majority of these patients (99–99.5%) will never progress to frank MDS or AML (6, 7). Since aging has been associated with a chronic inflammatory state, it is possible that clonal hematopoiesis is also promoted by inflammation (5, 8, 9). This could occur through increased genomic instability, which can lead to the acquisition of mutations, followed by the positive selection of mutant clones. It was recently shown in mouse models that hematopoietic stress, such as that induced by serial polyI-polyC injection, which activates toll-like receptor (TLR) signaling and induces HSCs to exit quiescence, can precipitate DNA damage in HSCs (10). This could be a potential mechanism for the increased acquisition of mutations in HSCs with age.

MDS-related mutations are the most commonly found in clonal hematopoiesis, including the “first hit” mutations that are thought to initiate clonality, such as those seen in genes that affect DNA methylation (TET2, DNMT3A) or histone acetylation (ASXL1) (11). The understanding of how epigenetic modifiers may regulate inflammatory signaling is evolving. Recent data suggest a causal link between some epigenetic mutations seen in clonal hematopoiesis or MDS and inflammation. In a study of over 17,000 blood samples from unselected patients, the patients with clonal hematopoiesis not only had a higher rate of hematologic malignancies but also a higher rate of death from cardiovascular disease and stroke compared to those without clonal hematopoiesis (7). TET2 (ten-eleven translocation-2) and DNMT3A (DNA methyltransferase 3A) are known to cause abnormalities in hematopoiesis, including in the monocyte-macrophage lineage, derangements of which are also seen in atherosclerotic disease and diabetes (7, 12). In fact, Jaiswal et al. recently showed that loss of *Tet2* in hematopoietic cells could promote atherosclerosis in the LDL-receptor knockout mouse model due to activation of macrophages (13). They found that macrophages from *Tet2−*/*−* bone marrow secreted increased levels of several chemokines, including CXCL1, CXCL2, CXCL3, PF4, and PBBP, some of which are known to promote atherogenesis. In patients with CHIP with TET2 mutations, they also found serum elevations of the inflammatory chemokine interleukin 8 (IL-8) (13). Another recent study also identified increased interleukin 1 beta (IL-1β) and inflammasome activation in mice with *Tet2* deficiency (14). Furthermore, Cull et al. found constitutive activation of the lipopolysaccharide (LPS)-related inflammatory pathway *in vivo* in peritoneal fluid in a *Tet2* knockdown mouse model, and increased IL-1β and interleukin 6 (IL-6) levels from bone marrow-derived macrophages *in vitro*, suggesting that chronic inflammation and dysregulation in the immune microenvironment is a result of Tet-2 loss (15).

Leoni et al. recently reported on the role of DNMT3A, another epigenetic modifier, in regulating mast cell inflammatory responses (16). They found that *Dnmt3a* knockout mast cells were more responsive to stimuli than wild-type mast cells, and secreted higher levels of inflammatory cytokines, such as IL-6, tumor necrosis factor alpha (TNF-α), and IL-13, leading to increased acute and chronic inflammatory responses *in vivo*. Together, these studies directly link inactivation of *Tet2* or *Dnmt3A*, two of the most commonly mutated genes in patients with clonal hematopoiesis or myeloid malignancies, with the initiation of an inflammatory state. Activation of inflammatory signaling can then lead to further expansion of mutant clones, by increasing cell cycling or enabling evasion from apoptosis, thereby promoting progression to MDS, MPN, and/or AML. Here, we discuss several mechanisms by which specific inflammatory signaling pathways can promote the clonal expansion of pre-LSCs and modulate disease progression, including pathways driven by interferon (IFN) I and II, TLRs, TNF-α, and ILs, in particular IL-1β, IL-6, and IL-8. Several of these pathways include potential therapeutic targets for the treatment or prevention of MDS, MPN, or AML.

## Type I IFNS (IFN-α/IFN-β)

**Figure 1:**
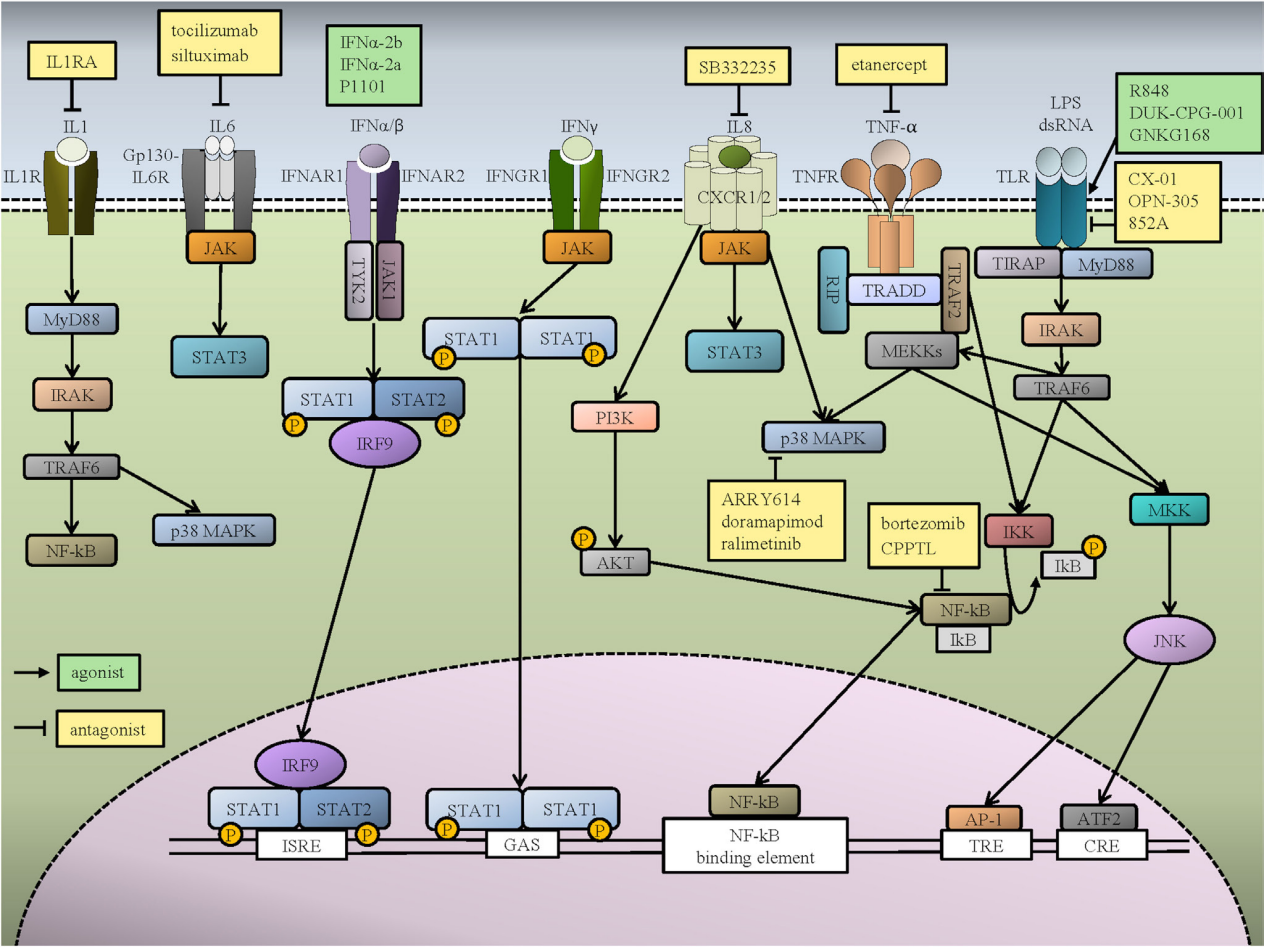
Inflammatory signaling pathways in hematopoietic cells and potential therapeutic targets for myeloid malignancies. Interleukin (IL)-1β activates the IL-1 receptor (IL-1R), which causes dimerization and intracellular downstream signaling *via* MYD88 and IRAK. This activates multiple downstream pathways, including NF-κB and p38 MAPK. Two interleukin 6 (IL-6) molecules form a hexamer with two IL-6 receptors (IL-6R) and two GP-130 molecules, which signal *via* the JAK1–STAT3 pathway. The binding of IFN-α/β to IFNAR receptors activates TYK2 and JAK1, which phosphorylate STAT1 and STAT2. The association of IRF9 and phosphorylated STAT1 and STAT2 activates transcription by binding to IFN-stimulated response elements (ISREs). IFN-γ binding to IFNGR receptors promotes STAT1 phosphorylation by JAK. The STAT1 homodimer translocates to the nucleus and activates IFN-γ-activated site (GAS) sequences. IL-8 binds to its receptor, either CXCR1 or CXCR2, which can activate various downstream signaling pathways, including PI3K/AKT, JAK/STAT, and MAPK. There is extensive crosstalk between tumor necrosis factor alpha (TNF-α) and Toll-like receptor (TLR) signaling pathways. TNF-α binds to its receptor TNFR and activates IKK *via* RIP and TRAF2 recruitment by TRADD. IKK activation promotes IKB phosphorylation and release of NF-κB, which can then translocate to the nucleus. TNF-α binding also activates p38 and MEKK. The activation of MEKK causes JNK to stimulate AP-1, which binds to TPA DNA-response elements (TRE) and ATF2, which binds to cAMP-responsive elements (CRE). Activation of TLR by infectious molecules initiates the signaling pathway through MyD88, which recruits IRAK to bind TRAF6 and activate NF-κB and JNK pathways. Representative pathways agonists (green boxes) and antagonists (yellow boxes) that are either in preclinical or clinical investigation are shown.

Interferons are known as key regulators of HSCs. They are categorized into type I IFN, including IFN-α and IFN-β, and type II IFN (also known as IFN-γ). Type I IFNs are produced endogenously in response to a program set by TLR3 activation in response to a variety of host challenges, such as viruses, and also to double stranded DNA from bacteria and tumors (17). Type I IFNs signal via the ubiquitously expressed IFN-α/β receptor (IFNAR, see Figure 1). IFNAR is composed of an extracellular heterodimer of two receptor tyrosine kinases, IFNAR2 (TYK2) and IFNAR1 (JAK1), whose binding leads to phosphorylation of STAT1 and STAT2, forming a trimer with unphosphorylated IRF9 (17). This trimer then enters the nucleus to direct transcriptional activity of a variety of antiviral cell programs, also leading to the production of the chemokine CXCL10 and induction of apoptosis pathways. IFN-α was initially tested as a therapy for chronic myelogenous leukemia (CML) in the 1970s, following *in vitro* studies showing inhibition of CML growth with IFN treatment. Subsequent clinical trial data showed up to a 60% complete cytogenetic response and improved overall survival compared to traditional chemotherapy. Rare complete long-term remissions post-IFN treatment were reported in a subset of patients who were treated without allogeneic stem cell transplantation (SCT), making this the standard of care for the treatment of CML prior to the era of tyrosine kinase inhibitors (TKIs) (18). IFN has also been used clinically in the treatment of Philadelphia chromosome (Ph)-negative MPNs, including polycythemia vera (PV), essential thrombocythemia (ET), and primary myelofibrosis (MF) (19, 20). While successful as the first biologic treatment in cancer, the mechanism of action of IFN-α in the treatment of MPN, or its effects on hematopoiesis in general, remained elusive.

One proposed mechanism of action for IFN-α in hematopoiesis is through activation of the p38MAPK-mediated apoptosis pathway. It was demonstrated that IFN-α treatment of KT-1 cells led to activation of p38 MAPK-mediated growth inhibition, which was reversed with p38 inhibitor treatment (21). Treatment of patient-derived CML cells grown in culture with IFN-α also showed decreased cell growth via upregulation of p38, and this effect was also reversed with a p38 inhibitor. Therefore, activation of p38 MAPK is likely to be one of the therapeutic effects of IFN-α in CML (21, 22).

Additional mechanisms of IFN-α action are likely to be important *in vivo*. Short-term IFN-α treatment induced most hematopoietic stem and progenitor cells (HSPCs) in mice to exit the quiescent state and enter active cell cycling (23). However, chronic IFN-α administration causes irreversible HSC dysfunction, as demonstrated by the inability to repopulate in a competitive repopulation assay. Furthermore, activation of IFN signaling in mice impairs hematopoietic recovery after 5-fluorouracil chemotherapy (23). Therefore, chronic activation of type I IFN signaling can deplete HSPCs by repeatedly driving them out of the quiescent state and impeding their return to steady state after activation.

Two studies using knock-in JAK2V617F murine models of PV suggest that IFN-α may play a similar role in MPN stem cells (24, 25). Both studies reported that HSPCs in the JAK2V617F model become more proliferative and lose quiescence after IFN-α treatment, leading to the depletion of MPN stem cells. Furthermore, IFN-α treatment can prevent disease initiation in secondary transplantation, suggesting that IFN-α treatment has a direct effect on MPN disease-initiating cells (24, 25).

## Clinical Applications of Modulating IFN-α/β Signaling

The proliferative effects of IFN-α on HSCs could have therapeutic utility in the treatment of myeloid malignancies, and this strategy is currently being explored in multiple clinical trials (Table 1). By inducing dormant LSCs to enter the cell cycle, IFN-α could make them more sensitive to chemotherapy or kinase inhibitors. Data from a few CML patients who were treated with IFN-α, followed by imatinib, shows that these patients achieved prolonged remissions, suggesting the depletion of pathologic LSCs with drug administration (26). Data from clinical studies in PV, ET, and MF patients show that treatment with IFN-α can cause cycling and differentiation of pathologic stem cells, and lead to a decrease of JAK2 allelic burden (9, 27). Similar results have been obtained in patients with calreticulin-mutated ET and MF (28). IFN treatment resulted in transfusion independence, decreased spleen size, and improved symptoms and quality of life in a significant proportion of MPN patients (29). There is also a strong rationale for combining IFN-α with JAK2 inhibitors in patients with JAK2-mutated MPNs (9, 25, 30). IFN-α is clinically approved for several indications, and there are numerous ongoing clinical trials that incorporate IFN into the treatment of MPN and AML, specifically in the post-transplant setting for relapse prevention (see Table 1). A recent interim report of one such clinical trial that examined the role of IFN-α in AML patients with minimal residual disease (MRD) after SCT demonstrated that 75% of the patients converted to MRD-negative status after IFN-α treatment (31). These promising early results suggest that IFN-α may be an effective approach in the postremission setting to eliminate LSCs or pre-LSCs in AML and in MPNs.

**Table 1:** Clinical trials targeting interferon α/β MDS, MPN, and AML.

Drug	Clinical trial	Status
IFN-α-2b	NCT03121079: IFN-α prevents leukemia relapse of AML patients after SCT	Phase I
IFN-α-2a	NCT02328755: PEG-INFα-2a to enhance antileukemic responses after allogeneic transplantation in AML	Phase I/II
IFN-α	NCT02027064: IFN-α for the intervention of molecular relapse in t (8;21) AML after allo-HSCT	Phase IV
IFN-α-2b	NCT02331706: IFN-DLI for relapsed acute leukemia after Allo-SCT	Phase I
IFN-α-2b	NCT00548847: Immunotherapy for AML, ALL, blast phase CML, and MDS, relapsed after allogeneic SCT	Phase II, completed
IL-12 + IFN-α	NCT00003451: IL-12 followed by IFN-α in treating patients with advanced cancer	Phase I, completed
IL-2 + IFN-α	NCT00002504: IL-2 plus IFN-α in treating adults with metastatic cancer (including leukemias, MDS, and MPN)	Phase II, completed
IL-2 + IFN-α	NCT00003408: Biological therapy (GM-CSF, interleukin 2, and IFN-α) following chemotherapy and SCT in treating patients with cancer (including MDS and MPN)	Phase II, completed
IFN-α-2a	NCT00452023: Pegasys^®^ in patients with MPNs	Phase II
IFN-α-2a	NCT02742324: Ruxolitinib and Peg-IFN-α-2a combination in patients with primary myelofibrosis RUXOPeg (RUXOPeg)	Phase I/II
PEG-proline-IFN-α-2b	NCT02370329: P1101 [polyethyleneglycol (PEG)-proline-IFN-α-2b] in treating patients with myelofibrosis	Phase II
PEG-proline-IFN-α-2b	NCT03003325: The benefit/risk profile of AOP2014 in low-risk patients with PV (low-PV)	Phase II
Nilotinib and PEG-IFN-α-2b	NCT02001818: Peg-IFN-α-2b and nilotinib for augmentation of complete molecular response in CML (PInNACLe)	Phase II
IFN-α-2b	NCT01657604: Tasigna and IFN-α Evaluation Initiated by the German CML Study Group—the TIGER Study (TIGER)	Phase III
IFN-α-2a	NCT02201459: Nilotinib ± Peg-IFN for first-line chronic phase CML patients (PETALs)	Phase III
PEG-proline-IFN-α-2b	NCT01933906: Addition of P1101 to imatinib treatment in patients with chronic phase CML not achieving a complete molecular response	Phase I
PEG-IFN-α-2a	NCT02381379: Malaysia stop tyrosine kinase inhibitor trial (MSIT): IFN-α vs. observation in CML patients off TKI after deep molecular remission × 2 years	Phase III

*Representative studies are listed. Source: www.clinicaltrials.gov*.

*IL, interleukin; MDS, myelodysplastic syndrome; MPN, myeloproliferative neoplasm; AML, acute myeloid leukemia; SCT, stem cell transplantation; HSCT, hematopoietic stem cell transplantation; CLL, chronic lymphocytic leukemia; CML, chronic myelogenous leukemia; PV, polycythemia vera; TKI, tyrosine kinase inhibitor*.

## Type II IFN (IFN-γ)

Type II IFN (also known as IFN-γ) activates the receptors IFNGR1 and IFNGR2, which signal through STAT1 (see Figure 1). It is produced by immune effector cells, such as NK and T-cells, and is important in the response to several intracellular pathogens, including some viral infections. The role of IFN-γ in HSCs is controversial [reviewed in Ref. (32)]. Originally, IFN-γ was demonstrated to inhibit the growth of human CD34+ cells, and to induce differentiation and apoptosis (33). However, more recent studies of both human and mouse HSCs have shown that IFN-γ can stimulate HSCs to proliferate, while promoting myeloid differentiation, suggesting that IFN-γ is important to maintain normal myeloid development in the setting of viral infections (34, 35). Subsequently, it was shown that only chronic IFN-γ exposure leads to HSC depletion (36).

Excessive IFN-γ signaling has been associated with hematopoietic dysfunction in humans. For instance, polymorphisms in the *IFNγ* gene have been linked with high production of IFN-γ, and occur more frequently in patients with aplastic anemia (37). Increased IFN-γ signaling has also been reported in MDS patients (38). In addition, previous studies have demonstrated the expansion of abnormal auto-reactive CD8 T cells in the bone marrow of MDS patients, which suggests a mechanism for increased production of myelosuppressive cytokines that affect hematopoietic cells in MDS (39–41). Furthermore, treatment with hypomethylating agents has been associated with elevated IFN-γ secretion in lower risk MDS (42). However, the detailed mechanisms through which IFN-γ promotes HSC incompetence in bone marrow failure syndromes are not well understood. To better understand the response to decitabine in MDS, Zhang et al. examined the levels of PD-L1, PD-1, and STAT1 in T-cells after decitabine therapy in lower risk MDS. They found that, although the level of STAT1 expression did not predict treatment response, an increase in the PD-1/STAT1 ratio was associated with hematopoietic improvement and prolonged survival in MDS patients treated with decitabine (43). Therefore, elevated IFN-γ/STAT1 signaling has also been associated with progression of MDS and treatment response.

Sharma et al. tested the engagement and functional role of protein kinase R (PKR) in the generation of IFN-γ effects on primitive hematopoietic progenitors and MDS cells. PKR is an IFN-inducible double-stranded RNA-activated serine-threonine protein kinase, which is a major mediator of the antiviral and antiproliferative activities of IFNs. Using a specific PKR inhibitor or siRNA-mediated PKR knockdown on bone marrow or peripheral blood mononuclear cells from MDS patients, they observed an increase in myeloid (CFU-GM), erythroid (BFU-E), and hematopoietic progenitor colony formation. Their data suggest that drugs that target PKR might be novel candidates for MDS therapy (44).

The role of IFN-γ in LSCs is still uncertain. To examine the role of IFN-γ in CML cells in the context of TKI treatment, Madapura et al. treated CML cell lines and primary human CML CD34+ cells with IFN-γ with and without imatinib. They showed that IFN-γ upregulates BCL6 *via* STAT1, as well as several antiapoptotic family members of the BCL2 family, including MCL-1L, the long isoform of MCL1. Interestingly, IFN-γ treatment also increased colony formation by CD34+ CML cells. These data support a protumorigenic effect of IFN-γ in CML and suggest that IFN-γ may contribute to TKI resistance. Their data suggest that combining TKIs with inhibitors of BCL6 or MCL1 is a potential approach to eradicate CML stem cells (45).

In contrast to the tumor-promoting effects of IFN-γ reported in MDS, Fatehchand et al. demonstrated that in AML, IFN-γ could induce cytotoxicity. IFN-γ treatment promotes myeloid differentiation of myeloblasts, and thereby potentiates the antibody-mediated cytotoxicity effect of daratumumab in several AML cell line-derived models. They also showed that IFN-γ treatment promotes the myeloid differentiation and phagocytic activity of primary AML patient cells. The combination of IFN-γ and FCγR activation enhanced the production of granzyme B, suggesting that IFN-γ can induce AML cells to differentiate into immune effector cells (46).

SOCS1 is an important negative regulator of IFN-γ signaling. The RIP1/RIP3 kinases, which are activated by TNF-α, inhibit the degradation of SOCS1, which limits the extent of IFN-γ signaling. Induction of RIP1/RIP3-mediated necroptosis has been proposed as an alternative strategy for treating apoptosis-resistant leukemia (47). In a recent study, Xin et al. demonstrated that, despite a high basal level of TNF-α secretion and RIP1/RIP3 signaling in the majority of French-American-British (FAB) subtype M4 and M5 AML samples, most AML cells do not undergo apoptosis. Using genetic and pharmacologic approaches, they showed that AML cells with inactivated RIP1/RIP3 signaling exhibit increased sensitivity to IFN-γ-induced differentiation, which leads to decreased clonogenic activity and apoptosis. Therefore, they suggested that the combination of IFN-γ with other inducers of differentiation could be a novel therapeutic strategy for AML (48).

## Clinical Applications of Modulating IFN-γ Signaling

While IFN-γ1β is an approved treatment for preventing infections in chronic granulomatous disease, and is currently being tested in multiple autoimmune conditions, solid tumors, and lymphoma, its use in myeloid malignancies has not yet been investigated in clinical trials. Because the roles of IFN-γ in pre-LSC and LSC function in MDS and AML are still unclear, further preclinical investigation with *in vivo* models is needed to better understand the appropriate clinical setting for targeting IFN-γ signaling in these diseases.

## TLR Signaling

Toll-like receptors are a family of pattern recognition receptors that are important in innate immunity. TLRs 1, 2, 4, and 6 transduce signals via the myeloid differentiation primary response gene 88 (MYD88), which leads to activation of IRAK1, 4, and 2, and TRAF6. This mediates an acute proinflammatory response through activation of the nuclear factor kappa-light-chain-enhancer of activated B cells (NF-κB), AP-1, and p38 MAPK pathways [reviewed in Ref. (49), see Figure 1]. TLRs are expressed on HSPCs, effector immune cell populations, and stromal cells (50–52). Several classes of TLRs, including TLR4, TLR7, TLR8, and TLR9, are expressed on human CD34+ cells, suggesting that HSCs have an early mechanism to immediately detect infection (32). Although normal TLR signaling plays an important role in the immune response to injury or infection, enhanced or abnormal TLR signaling has been linked with defective hematopoiesis and hematopoietic malignancies (53, 54). Takizawa et al. recently demonstrated that LPS, the best known ligand for TLR signaling, can directly stimulate HSCs *in vivo* and increase their cycling. They showed that, while LPS induces the proliferation of dormant HSCs, prolonged LPS exposure impairs HSC regenerative capacity (55). They also found that this process is mediated *via* the TLR4-TRIF-ROS-p38 pathway, and not through MyD88 signaling. This suggests that inactivation of the TRIF-ROS-p38 signaling axis could prevent the induction of HSC dysfunction by LPS, while not affecting emergency myelopoiesis (55).

In addition, LPS increases the number of colony forming units in the blood, spleen, and bone marrow (56, 57). Activating mutations, increased expression of TLRs and TLR signaling pathway intermediates, and loss of repressors of TLR signaling have all been reported in MDS (49, 58–64). Interestingly, while increased expression of TLR2 and TLR9 has been reported in MDS, their expression decreases with progression to AML (49). This suggests that TLRs may play different roles in pre-LSCs in MDS and in LSCs in AML.

Recent studies have shed more light onto the mechanisms of activation of TLR signaling in del (5q) MDS. The S100 calcium-binding protein family members S100A8 and S100A9, which are released during the activation of phagocytes, have been described as endogenous activators of TLR4 (65). Schneider et al. have linked S100A8 and S100A9 to HSC dysfunction and impaired erythroid differentiation in del (5q) MDS. They showed that impaired erythropoiesis in del (5q) MDS is associated with heterozygous deletion of RPS14 (ribosomal protein small subunit 14). Using conditional knockout Rps14 mice, they detected a p53-dependent defect in erythroid differentiation. This differentiation defect resulted in age-dependent progressive anemia, megakaryocyte dysplasia, and loss of HSC quiescence. Using proteomic profiling, they observed a higher level of S100A8 and S100A9 expression in Rps14 mutant erythroblasts. By genetically inactivating S100A8 expression they rescued the erythroid differentiation defect in Rps14 haploinsufficient HSCs (66). This suggests that secretion of S100A8 and S100A9 by activated immune effector cells could promote hematopoietic dysplasia *via* TLR signaling.

Starczynowski et al. have shown that, in del (5q) MDS patients, loss of miR-145 and miR-146a can also cause the abnormal activation of TLR signaling (62). These micro-RNAs normally inhibit the TLR signaling intermediates TIR-domain-containing adaptor protein and TRAF6, a TLR effector with ubiquitin (Ub) ligase activity. Moreover, overexpression of TRAF6 or knockdown of miR-145 or miR-146 mimics some of the features of del (5q) MDS in mouse models, suggesting that aberrant activation of TLR pathway signaling in HSCs contributes to disease pathogenesis (62, 67).

It has also been shown previously that TLR-driven pathways are involved in coordinating RNA processing during hematopoietic differentiation (68, 69). Fang et al. demonstrated the role of TRAF6 in RNA processing in hematopoietic cells by examining RNA ubiquitination. Using a global ubiquitination screen, they identified hnRNPA1, an RNA-binding protein and auxiliary splicing factor, as a substrate of TRAF6. TRAF6-mediated ubiquitination of hnRNPA1 regulates the alternative splicing of *Arhgap1*, which activates the GTP-binding Rho family protein Cdc42, and contributes to the HSPC dysfunction observed in the TRAF6 overexpression mouse model (67).

Varney et al. investigated the role of the TRAF-interacting protein with forkhead-associated domain B (TIFAB) in MDS, which is a haploinsufficient gene in del (5q) MDS. Loss of heterozygosity of TIFAB causes bone marrow failure and significant changes in myeloid differentiation. Gene expression analysis in TIFAB knockout HSPCs revealed the upregulation of immune and infection response signatures, which suggests hypersensititivy to TLR4 stimulation. However, TNF and endotoxin signatures were downregulated. Using a global proteomic analysis, this study revealed that TIFAB forms a complex with TRAF6 and decreases the stability of TRAF6 *via* a lysosome-dependent mechanism. Therefore, loss of TIFAB increases TRAF6 protein levels and thereby activates NFKB, which leads to ineffective hematopoiesis. Furthermore, the authors observed that deletion of both TIFAB and miR-146a increases the expression of TRAF6, suggesting that these factors cooperate in promoting dysfunctional hematopoiesis (70).

Recent work has further highlighted the importance of TLR signaling in the bone marrow microenvironment in the initiation of preleukemic disorders. Using the preleukemic Shwachman-Diamond syndrome (SDS) mouse model driven by deletion of the *Sbds* gene in mesenchymal progenitor cells, Zambetti et al. have shown that *Sbds* deletion drives mitochondrial dysfunction, oxidative stress, and activation of the DNA damage response in HSPCs. The authors performed RNA sequencing of purified mesenchymal cells from SDS mice and also of sorted mesenchymal cells from patients with three preleukemic diseases: SDS, low-risk MDS, and Diamond-Blackfan anemia. When comparing the overexpressed genes in each case, they identified the p53-S100A8/9-TLR inflammatory signaling pathway as a common driving mechanism of genotoxic stress in these diseases. Remarkably, they also demonstrated that overexpression of S100A8 and S100A9 in mesenchymal cells is sufficient to induce DNA damage and apoptosis in wild-type HSPCs in a paracrine manner via activation of TLR signaling. Furthermore, S100A8/9 expression in mesenchymal cells, associated with activated p53 and TLR signaling, predicted leukemic evolution and decreased progression-free survival in low-risk MDS patients (71). Since MDS is a heterogeneous disease with a variable prognosis (1), this finding could have significant clinical relevance. This study provides strong evidence that TLR signaling may play an important role in the premalignant microenvironment, which promotes HSPC dysfunction, and leads to the generation of pre-LSCs in MDS and other bone marrow failure disorders. However, none of the mice in this study developed AML, possibly because intrinsic HSPC factors likely also play a role in disease progression.

Dimicoli et al. have recently demonstrated that MYD88, a key mediator of TLR innate immune signaling, is potentially involved in the pathogenesis of MDS. While this study did not find any mutations in MYD88 in MDS, they detected higher expression of MYD88 in 40% of MDS patient cells compared to normal CD34+ cells. MYD88 blockade caused increased erythroid colony formation, and suppressed the secretion of IL-8. They concluded that MYD88 mediates innate immune signaling in MDS, and that inhibition of MYD88 could potentially improve erythropoiesis in this disease (63).

While TLR signaling has been implicated in the emergence of pre-LSCs in MDS, TLR signaling in AML appears to play a different role. Ignatz-Hoover et al. found that resiquimod (R848), a TLR7/8 agonist, promotes the differentiation of AML blasts in a TLR8/MyD88/p38-dependent manner. They also observed antileukemic activity of R848 in a xenograft mouse model of AML (72). Furthermore, Zhong et al. showed that combining R848, LPS, and TNF-α or the combination of TNF-α, and R848, caused significantly higher cytotoxicity to AML cells than TNF-α or R848 alone (73). These data suggest that stimulating TLR8, particularly in combination with other inflammatory signaling pathways, can offer a potential therapeutic strategy for AML (72).

The S100A8 and S100A9 calcium-binding proteins are also highly expressed in AML, and their expression has been linked to poor prognosis in this disease (74). To investigate the roles of S100A8 and S100A9 in AML, Laouedj et al. examined their protein expression in two mouse models of AML and in AML patient samples. They found that S100A8/A9 are secreted by leukemic blasts, and not by the microenvironment. While S100A proteins were not required for AML initiation in the HoxA9-Meis1 mouse model of AML, treatment with an anti-S100A8 antibody induced AML cell differentiation *in vivo* and impaired AML progression. Interestingly, treatment with recombinant S100A9 protein prolonged survival in the same mouse model of AML, suggesting an antagonistic relationship between S100A8 and S100A9. Investigating the pathways involved in S100A9-induced AML cell differentiation revealed that S100A9 induces differentiation *via* TLR4 and several downstream factors, including p38 MAPK, extracellular signal-regulated kinases 1 and 2 (ERK1/2), Jun N-terminal kinase (JNK), and NF-κB. The authors concluded that S100A9 induces differentiation of AML, while S100A8 prevents S100A9-induced differentiation, and that the ratio of S100A9 to S100A8 determines the degree of differentiation in AML (75).

## Clinical Applications of Modulating TLR Signaling

While a great deal of preclinical evidence supports that activation of TLR signaling promotes the emergence of pre-LSCs in MDS, the clinical utility of inhibiting TLR signaling in MDS is unclear. Several clinical trials have focused on inhibiting TLRs in hematologic malignancies (Table 2). The humanized anti-TLR2 antibody is being studied in a phase I/II study as a second-line treatment for lower risk MDS (NCT02363491). In addition, the TLR2/4 antagonist CX-01 is being tested in a phase I trial for relapsed/refractory MDS and AML in combination with azacitidine (NCT02995655).

**Table 2:** Clinical trials targeting inflammatory signaling pathways in MDS, MPN, and AML.

Target	Drug	Mechanism	Clinical trial	Status
TLR	CX-01	Inhibitor of TLR2 and TLR4	NCT02995655: CX-01 combined with azacitidine in the treatment of relapsed refractory MDS/AML	Phase I
TLR	OPN-305	Humanized anti-TLR2 antibody	NCT02363491: A phase I/II study of OPN-305 as second line in lower risk MDS	Phase I/II
TLR	DUK-CPG-001	TLR9 agonist	NCT02452697: Phase II NK cell-enriched DLIs with or without DUK-CPG-001 from donors following allogeneic SCT (NK-DCI)	Phase II
TLR	GNKG168	Oligonucleotide that acts as TLR9 agonist	NCT01743807: Phase I study of GNKG168 in pediatric acute lymphoblastic leukemia (ALL) and AML	Phase I, terminated
TLR	852A	TLR7 agonist	NCT00276159: Phase II study of 852A administered subcutaneously in patients with hematologic malignancies not responding to standard treatment (76)	Phase II, completed
p38-MAPK	ARRY614	Inhibitor of p38 MAPK and Tie2	NCT0149649: Hematological improvement in lower risk MDS patients who previously failed azanucleoside treatment (77)	Phase I, completed
IL-6	Tocilizumab	Anti-IL-6 antibody	NCT02057770: Allogeneic or haploidentical SCT followed by high-dose cyclophosphamide in treating patients with relapsed or refractory AML	Phase I
IL-6	Siltuximab	Anti-IL-6 antibody	NCT02805868: Siltuximab in treating patients with primary, post-PV, or post-ET MF	Phase I, withdrawn
IL-6	Siltuximab	Anti-IL-6 antibody	Phase II study comparing siltuximab plus best supportive care (BSC) with placebo plus BSC in anemic patients with IPSS low- or int-1-risk MDS (78)	Phase II
TNF-α	Etanercept	IgG inhibitory antibody against TNFR	NCT00118287: Azacitidine and etanercept in treating patients with MDS	Phase I/II, completed
NF-κB	Bortezomib	Proteasome inhibitor inhibits NF-κB	Phase I study of bortezomib in combination with idarubicin and cytarabine in patients with AML (79)	Phase I, completed
NF-κB	Bortezomib		Phase I study using bortezomib with weekly idarubicin for treatment of elderly patients with AML (80)	Phase I, completed
NF-κB	Bortezomib		NCT00262873: Bortezomib in treating patients with MDS	Phase II, completed
NF-κB	Bortezomib		Phase II study of bortezomib combined with chemotherapy in children with AML (81)	Phase II, completed

*Representative studies are listed. Source: www.clinicaltrials.gov*.

*IL, interleukin; MDS, myelodysplastic syndrome; MPN, myeloproliferative neoplasm; AML, acute myeloid leukemia; TLR, toll-like receptor; DLI, donor lymphocyte infusion*.

In contrast, another emerging approach in clinical trials is to use TLR agonists to induce differentiation in MDS and AML (Table 2). Weigel et al. performed a phase II study on the TLR7 agonist imidazoquinoline (852A) on patients with recurrent hematologic malignancies, including six AML patients. They assessed the activity of 852A when administered with prolonged dosing and its safety and ability to activate the immune system. They observed that 852A can be safely administered twice weekly with prolonged tolerability. However, only one partial remission was observed in this small cohort of AML patients (76). Another phase I trial (NCT01743807) tested the oligonucleotide GNKG163, which acts as a TLR9 agonist, in acute lymphoblastic leukemia (ALL) and AML with MRD, but the trial was terminated. Because the roles of TLR signaling in MDS and AML are complicated, and largely dependent on disease stage and clinical context, future design of clinical trials incorporating TLR-modulating agents will need to take into account these context-dependent effects.

## IL-1β

Interleukin 1 beta is the first of 11 ILs of the IL-1 family, a proinflammatory cytokine produced by myeloid cells in response to TLR stimulation by infection and by non-infectious stressors. IL-1β is activated by caspase-1, which activates the IL-1R1 receptor, causing dimerization with IL-1 accessory protein (IL-1RAP). This leads to the dimerization of intracellular TIR complexes, which then engages the MYD88-IRAK4 complex, thereby activating multiple downstream pathways, including NF-κB and p38 MAPK, in multiple organ systems (see Figure 1) (82). IL-1β is an important stromal growth factor in the maintenance of multipotent mesenchymal stromal cells and enhances the ability of stromal cells to maintain HSCs, as shown in both *in vitro* mouse studies and long term culture-initiating cell assays on human cells (83, 84). A study by Pietras et al. elucidated the function of IL-1β in normal HSC *in vivo*. They found that short-term or acute IL-1β administration caused rapid myeloid differentiation of HSCs, and also facilitated recovery of the myeloid lineage after 5-FU myeloablation *via* activation of NF-κB, which leads to activation of the myeloid transcriptional program orchestrated by the transcription factor PU.1(85). However, chronic or long-term administration of IL-1 caused decreased competitive repopulation activity, suggesting a replication challenge and impaired self-renewal of HSCs. This effect was reversible upon withdrawal of IL-1 (85). Furthermore, Hérault et al. also showed that IL-1 signaling in HSPCs, induced by IL-1 secreted by the bone marrow niche after 5-FU treatment, is required for myeloid regeneration (86). However, IL-1β cannot induce differentiation of human HSC *in vitro* (87). Therefore, it appears that IL-1β is required but not sufficient for normal myeloid differentiation in the setting of myeloablation, but that chronic administration of IL-1 impairs normal HSPC function.

Several recent studies have elucidated the functional role of IL-1β in myeloid malignancies. IL-1β levels are elevated in the serum of patients with several preleukemic and leukemic conditions, which makes IL-1β a potential therapeutic target [reviewed in Ref. (82)]. In Ph-MPNs such as PV and PMF, high IL-1 levels are associated with a worse prognosis, and may suggest a higher likelihood of progression to fibrosis (88). In CML, levels of IL-1 and IL-1RAP are elevated, seen more often in blast crisis, and predict a poor prognosis (89). In a murine model of MPN driven by JAK2-V617F, it was observed that MPN stem cells secrete IL-1β, which induces mesenchymal stem cell (MSC) death and resultant disease expansion (90). This effect on the bone marrow microenvironment was partially reversible with IL-1β-inhibitor treatment (90). Hérault et al. showed that the preleukemic niche in two different mouse models of MPN secretes elevated levels of IL-1, which drives the differentiation of HSPCs into proliferative granulocyte/macrophage clusters (86). In CML, Zhang et al. demonstrated that LSCs have increased expression of IL-1 receptors and IL-1RAP and that treatment with an IL-1 receptor antagonist (IL-1RA) in a CML mouse model inhibits IL-1 signaling and growth of CML LSCs *in vivo* (91). Furthermore, they showed that IL-1RA cooperates with TKIs in the elimination of CML LSCs. Therefore, IL-1β is a promising therapeutic target in MPNs.

The role of IL-1β in AML is more complicated. Several previous studies have shown that IL-1β is expressed by AML blasts, associated with poor prognosis, and promotes the proliferation of AML blasts [reviewed in Ref. (82)]. A recent study by Katsumura et al. elucidates how IL-1 might become upregulated in AML. They found that p38 MAPK and MEK1 induce hyperphosphorylation of the master HSC transcription activator GATA-2 in human AML cell lines (92). This leads to increased expression of IL-1β, which is a transcriptional target of GATA-2. Because IL-1β activates p38 MAPK, this produces a p38-GATA-2-IL-1β positive feedback loop (92). They also observed a correlation between GATA-2 and IL-1β expression levels in AML patients. Furthermore, they observed that a higher IL-1 level in the bone marrow of AML patients portends a poor prognosis (92). However, serum data from AML patients in other studies showed a 10-fold lower level of IL-1 expression compared with normal controls (93). The different subtypes of AML examined in these two studies could partially account for these contradictory results regarding IL-1 expression levels. Katsumura et al. found higher IL-1 expression in M4-M5 AML, while Su et al. focused on patients with M0-M2 AML (92, 93).

Interestingly, Yang et al. found that expression of IL-1β was downregulated in the CD34+CD38− LSC-enriched population of AML cells compared with mature blasts and normal CD34+ cells, and that lentiviral expression of IL-1β in these cells inhibited their self-renewal and promoted cell cycle progression (94). However, low dose IL-1β exposure in the same study could stimulate colony formation by AML cells (94). In contrast, Carey et al. recently identified IL-1β in a functional screen as one of the factors that promoted the growth of patient AML cells *ex vivo*, while suppressing the role of normal HSPCs. They found that IL-1β is mostly produced by macrophages, and its levels are elevated in the serum of AML patients, most consistently in patients with the FAB M4 or M5 subtypes of AML. Furthermore, siRNA knockdown of IL-1R1 decreased the proliferation of AML blasts, and genetic deletion of *il1r1* prolonged survival in a murine model of AML driven by AML1-ETO9a and Nras^G12D^. They also found that IL-1β-sensitive AML samples have increased phosphorylation of p38-MAPK, and that the p38-MAPK inhibitors doramapimod (BIRB-796) or ralimetinib could inhibit the IL-1β-dependent growth of AML patient mononuclear cells or AML CD34+ cells. Interestingly, treatment of normal CD34+ cells *ex vivo* with doramapimod also rescued the inhibitory effects of IL-1β, suggesting that treatment with p38-MAPK inhibitors could improve normal hematopoiesis while inhibiting leukemic growth (95).

Carter et al. demonstrated that coculture of several human AML cell lines with MSCs could lead to an increase in IL-1β expression by AML cells, and that *in vitro* inhibition of IL-1β by IL-1βRA in cultured OCI-AML3 cells suppressed leukemic cell migration and sensitized to cytarabine chemotherapy (96). In MLL-rearranged AML, Liang et al. showed that IL-1 signaling promotes degradation of the wild-type MLL protein *via* phosphorylation of the ubiquitin ligase UBE20 (97). Inhibiting the degradation of wild-type MLL using IRAK1/4 and IRAK4 inhibitors increased the stability of wild-type MLL in MLL-AF9 AML, which displaces the MLL fusion protein from some of its chromatin targets and leads to deregulation of the gene regulatory network in MLL-rearranged AML. As a result, IRAK1/4 inhibitor treatment caused increased survival and impaired LSC function in the MLL-AF9 mouse model of AML (97). This novel mechanism could account for some of the activity of IL-1 pathway inhibitors reported in other studies in secondary AML, and specifically in MLL-AF9 AML (98). Together, these results suggest that there may be differences in how leukemic blasts and LSCs regulate IL-1β expression and respond to IL-1β, and that there may be differential dose-dependent effects of IL-1β on both normal HSPCs and LSCs. In MPN and in at least some subtypes of AML, it is clear that IL-1β promotes the proliferation and maintenance of LSCs. However, the role of IL-1 signaling in the emergence of pre-LSCs and in disease progression to AML is less clear.

## Clinical Applications of Modulating IL-1β Signaling

Overall, inhibition of the IL-1 signaling pathway appears to be a promising approach to the treatment of myeloid malignancies. Multiple potential therapeutic agents that target IL-1 signaling have been tested preclinically in myeloid malignancies, but only a few are currently under clinical investigation. The IL-1R1 receptor antagonist IL-1RA, a competitive inhibitor of IL-1α and IL-1β, has preclinical activity in JAK2-V617F positive MPNs and CML (82, 91). IRAK1/4 inhibitors also have preclinical activity in MLL-rearranged AML (97). There have also been preclinical studies using antibodies to target IL-1RAP in CML and AML, but in many cases the mechanism of action is to eliminate IL-1RAP-expressing leukemic cells, rather than direct inhibition of IL-1 signaling (99–101). In addition, the IL-1β-specific blocking antibody canakinumab and the IL-1 receptor antagonist anakinra are both approved by the FDA for the treatment of some inflammatory disorders, but have not yet been studied clinically in patients with hematologic malignancies. Finally, the p38 MAPK inhibitor ralimetinib, which had preclinical activity in reducing the proliferative effects of IL-1 in AML, is in clinical trials for ovarian cancer, but has not been tested clinically for hematologic malignancies (95). Given the potential dose-dependent effects of IL-1β on both normal HSPCs and LSCs, and the differences in IL-1 expression among AML subtypes, the design of clinical trials to target IL-1 signaling may be challenging.

## Interleukin 6

Interleukin 6 is a proinflammatory cytokine that is released by monocytes and macrophages as part of the acute phase response to viruses and bacteria, in response to signals such as TLR, IL-1, and TNF, and by T cells during chronic inflammation. This leads to the recruitment of neutrophils to sites of injury (102, 103). The effects of IL-6 can be mediated by binding to the IL-6 receptor on the cell membrane (the classical signaling pathway, as in acute inflammation) or by binding to a soluble IL-6 receptor (causing a trans-signaling pathway that is believed to cause chronic inflammation). In both cases, a hexamer is formed with two IL-6 molecules, two IL-6 receptors (either membrane bound or soluble) and two membrane-bound gp130 molecules, which cause identical downstream effects via the JAK1–STAT3 pathway (see Figure 1) (102, 103). In embryonic HSC development, IL-6 mediates the increased production of HSCs, working downstream of the HIF1α/PGDFRβ signaling pathway, but may also respond to hypoxia independently as a proinflammatory cytokine (104). IL-6 is also produced by adult HSCs in response to stressors, including sepsis, chemotherapy, or in the post-transplant setting, and promotes myelopoiesis (105). Chronically increased IL-6, as seen in inflammatory conditions, has been shown to decrease hemoglobin production in late stage erythroid precursors and causes direct mitochondrial impairment (102). This is one of the likely etiologies of anemia of chronic inflammation. In addition, aged MSCs secrete IL-6, which disrupts their crosstalk with HSCs and impairs HSC quiescence in the bone marrow (106).

Reynaud et al. have shown that IL-6 also plays an important role in pre-LSCs and LSCs. In a murine model of CML driven by BCR-ABL expression in HSCs, they demonstrated that CML is induced and sustained by high IL-6 levels produced by BCR-ABL-expressing cells (107). This leads to expansion not only of BCR-ABL mutated cells, but also of wild-type hematopoietic cells, which accelerates disease progression in a paracrine fashion. The dysfunction of normal HSCs in response to high IL-6 levels in this CML model can be rescued with an anti-IL-6 antibody (108). Furthermore, studies with CML patient samples also confirmed the importance of IL-6 in promoting the proliferation and differentiation of CML cells (108). These studies highlight the importance of the proinflammatory changes in the microenvironment induced by leukemic cells, and suggest that IL-6 could be an important component of that pathologic microenvironment. In several different mouse models of Ph-MPNs, including MF driven by MPL-W515L or JAK2-V617F, single cell analysis showed high expression of IL-6, which was most highly expressed from mutated stem cells, but also expressed by wild type stem cells, suggesting an important role for IL-6 in the pathogenesis of Ph-MPNs (109).

Elevated IL-6 levels have been observed in many patients with preleukemic and leukemic conditions, though its predictive value as a biomarker is unclear. In patients with JAK2-V617F PV and PMF, the levels of several inflammatory cytokines were elevated, including IL-6 and IL-8 (110). Furthermore, Reikvam et al. showed that co-culture of patient-derived AML cells with healthy donor-derived MSCs led to increased IL-6 secretion into the media (111). In addition, Lopes et al. found that IL-6 levels in MSCs correlate with disease progression from MDS to AML, with only slight IL-6 elevations in the MSCs of MDS patients, and higher levels in MSCs from AML patients (112). However, another study found that MDS patient samples have increased IL-6 levels compared to healthy controls, but this does not correlate with disease stage (113). Elevated serum IL-6 levels have also been reported in AML patients compared to healthy controls (93, 114). These studies suggest that IL-6 may have a role in promoting the progression of MPN and MDS to AML, but more functional preclinical data are needed to better understand its role in preleukemic stem cells and LSCs.

Interestingly, Zhang et al. found that Tet2 represses the transcription of IL-6 in dendritic cells and macrophages during inflammation. They showed that Tet2 mediates this repression by recruiting histone deacetylase 2 (HDAC2) to prevent constant transcriptional activation of IL-6 in response to inflammation (115). This suggests that targeting Tet2/Hdac2-mediated gene-specific repression could be a novel therapeutic approach to decrease IL-6 signaling in patients with hematologic malignancies.

## Clinical Applications of Modulating IL-6 Signaling

The anti-IL-6 antibody tocilizumab, which is approved by the FDA for use in several rheumatologic diseases, is currently being tested for its possible anti-inflammatory effects in haploidentical SCT (NCT02206035). However, there are currently few clinical trials testing IL-6 inhibitors in hematologic malignancies (see Table 2). A phase II double blind randomized controlled study of siltuximab, an anti-IL-6 inhibitor, studied in transfusion-dependent low risk MDS, was terminated early due to futility (78). IL-6 levels may also be affected by several kinase inhibitors that are currently in clinical use for hematologic malignancies, such as ruxolitinib, a JAK inhibitor in the treatment of MF and PV (109). The clinical significance of elevated IL-6 levels in myeloid malignancies, both as a predictive biomarker and as a therapeutic target, needs to be further elucidated.

## IL-8

Interleukin-8 is a proinflammatory chemokine that is released in response to IL-1 or TNF-α as a result of environmental stressors such as infection, hypoxia and chemotherapy, and can act as a neutrophil chemoattractant. IL-8 promotes homing of neutrophils to the site of injury, entrapment and killing of bacteria by promoting neutrophil extracellular traps, phagocytosis, and oxidative burst, and also facilitates healing *via* angiogenesis (116, 117). IL-8 binds to one of its two G protein-coupled receptors, CXCR1 or CXCR2. These receptors are often present on endothelial and myeloid lineage cells, but can also be present on tumor cells (see Figure 1) (116). Once coupled with its receptor, the IL-8 program signals *via* activation of several downstream pathways, including PI3K/AKT, PLC/PKC, MAPK, FAK, and JAK/STAT (117). The CXCL8 gene that encodes IL-8 is not expressed in rodents, so its role in hematopoiesis cannot be studied through genetic inactivation in murine models. Therefore, the understanding of the roles of IL-8 in HSC function is limited.

However, emerging data suggest that IL-8 plays an important role in hematologic malignancies. Serum IL-8 levels were found to be increased in MDS and also in PV and ET, independent of JAK2V617F mutation status (118). In CML patients, it was reported that high serum IL-8 levels with low serum TGFβ3 could predict treatment outcome better than the traditional Sokal score (119). In addition, Schinke et al. reported that IL-8 and its receptor CXCR2 are expressed at higher levels in pre-LSCs from patients with MDS than in normal human CD34+ cells (120). Furthermore, knockdown or pharmacologic inhibition of CXCR2 with the inhibitor SB332235 in AML cell lines and in MDS and AML patient samples led to G0/G1 cell cycle arrest, and also inhibited leukemia progression in a xenograft mouse model (120).

Corrado et al. showed that exosomes secreted by CML cells stimulate the bone marrow microenvironment to produce IL-8, which in turn promotes survival of a CML cell line *in vitro* and in a xenograft mouse model *in vivo* (121). In a coculture study with AML patient samples, IL-8 was secreted by the bone marrow microenvironment as a result of hypoxia (O_2_ 1% for 48 h) by AML cells more than by normal cells (122). Among AML subtypes, acute promyelocytic leukemia had the lowest levels of IL-8 secretion, while FLT3-ITD AML had the highest IL-8 levels, which can predict for poor prognosis in FLT3-ITD AML (122). Abdul-Aziz et al. also demonstrated that AML cells cocultured with bone marrow stromal cells secrete macrophage inhibitory factor, which stimulates IL-8 production by the stroma, which in turn promotes the survival of AML cells (123). Furthermore, shRNA knockdown of IL-8 inhibited the prosurvival effects of the stroma on AML cells (123). Cordycepin, an adenosine analog, blocks mesenchymal stromal/stem cells from expressing VCAM-1 or IL-8 via impaired NF-κB signaling. The inhibitory effects of cordycepin in preclinical AML models support the importance of targeting the crosstalk between AML cells and the bone marrow niche in the treatment of AML. Combined with an adenosine deaminase inhibitor, cordycepin prolonged survival in U937 and K562 xenograft mouse models of AML (124). While IL-8 could be useful as a biomarker in multiple hematologic malignancies and could be a promising therapeutic target for MDS and AML, no clinical trials have been initiated targeting IL-8 or its downstream mediators.

## TNF-α/NF-κB Pathway

TNF-α is a major proinflammatory cytokine produced by macrophages upon stimulation with endotoxin or bacterial antigens. TNF-α signaling is mediated through the p55 receptor (TNFRSF1A), which is expressed on all nucleated cells and the p75 receptor (TNFRSF1B), which is only present on hematopoietic cells (see Figure 1) (32). The roles of TNF signaling in HSCs are controversial [reviewed in Ref. (32)]. While baseline TNF signaling is known to be important for normal HSC maintenance, excessive TNF-α signaling is associated with bone marrow failure and MDS (32). TNF-α stimulates NF-κB, which has a well-described role in malignancy (125–128). The important roles of TNF-α and NF-κB in MDS have been extensively reviewed elsewhere (129), so we will focus on more recent studies describing their roles and regulation in pre-LSCs and LSCs in MDS and AML.

It has been previously reported that TNF-α is upregulated in the bone marrow plasma and peripheral mononuclear cells of MDS patients, and is positively correlated with apoptosis in early stage/low risk MDS (129). Hence, TNF-α upregulation can play a crucial role in the impairment of hematopoiesis during MDS progression. To uncover the mechanism of TNF-α elevation in hematopoietic malignancies, Shikama et al. studied the expression of c-Fos under the regulation of its targeting miRNAs, miR-34a and miR-155. They demonstrated a significant decrease in stability of c-Fos mRNA as a consequence of miR-34 overexpression in AML cells. Higher levels of miR-34a expression in the blood of MDS patients correlated with increased TNF-α overexpression in granulocytes upon LPS stimulation (130).

Several recent studies have implicated TNF signaling in myeloid LSC function. In a study of the TNF superfamily ligand-receptor pair CD70/CD27 in AML, Riether et al. found that AML blasts and AML stem/progenitor cells express both CD70 and CD27. Moreover, soluble CD27 is expressed at significantly higher levels in the sera of newly diagnosed AML patients than in healthy controls, and can be used as a strong prognostic biomarker for survival. They also demonstrated that blocking the CD70/CD27 interaction with an anti-CD70 monoclonal antibody leads to increased differentiation and survival in a patient-derived AML xenograft model, including in secondary transplantation experiments without additional treatment. This suggests that the CD27/CD70 interaction is also important for LSC function. On the other hand, HSPCs from healthy human bone marrow did not express CD70/CD27, and were not affected by antibody treatment. Therefore, blocking the interaction between CD70/CD27 could be a novel therapeutic strategy to inhibit TNF signaling in AML with potential for a good therapeutic window (131).

Zhou et al. demonstrated that the trans-membrane form of TNF-α (tmTNF-α) is expressed specifically on LSCs in AML and ALL, using a monoclonal antibody termed C1, which specifically recognizes tmTNF-α and not its secretory form (132). They also found that leukemia cells are more sensitive to chemotherapy *in vitro* after tm-TNF-α knockdown, and that tm-TNF-α inhibition with the C1 antibody delays the onset of leukemia in patient-derived AML xenografts. Importantly, they demonstrated that treatment with the C1 antibody in primary transplant mice led to reduced engraftment of leukemic cells and a decreased disease burden, supporting the important role of tmTNF-α in LSCs *in vivo*. Additionally, they showed that *in vivo* targeting tmTNF-α with C1 antibody does not affect normal hematopoietic cells, suggesting a favorable therapeutic window for the use of this antibody in patients (132).

NF-κB is a transcription factor that is well known as an important regulator of cell survival, proliferation, and differentiation. It is both activated by TNF-α signaling, and can also regulate the expression of TNF-α (133–135). Although the activity of NF-κB is not detectable in normal unstimulated CD34+ HSCs, and NF-κB levels were reported to be low in low-risk MDS, they are increased in high-risk MDS patients, and correlate with increased blast counts, suggesting a role for NF-κB in the transition from pre-LSCs to LSCs (136). The activity of NF-κB has been observed in several molecular subtypes of AML, and it is likely that this pathway is involved in the progression to AML (137). Furthermore, it has been shown that NF-κB is highly expressed in primitive CD34+CD38− cells in AML, the population that is enriched in LSCs (138–140).

It has been demonstrated that inhibition of NF-κB can effectively eradicate LSCs while sparing normal HSPCs (138, 141). However, *in vivo* inhibition of NF-κB cannot completely eliminate AML cells, indicating that there are parallel survival signals in leukemic cells. To identify compensatory survival signals for NF-κB inhibition, Volk et al. demonstrated that AML stem and progenitor cells can be sensitized to NF-κB inhibition by inhibiting TNF-JNK signaling (142). They also reported that in some subtypes of AML, including M3, M4, and M5, leukemic cells produce endogenous TNF-α, leading to an increase in proliferation and survival of AML blasts through an autocrine mechanism *via* downstream signaling through both NF-κB and JNK-AP1 (142).

Recently, Li et al. reported that CD34− leukemic blasts in M4 and M5 AML also secrete IL-1β, while more immature CD34+ cells primarily secrete TNF-α, and that IL-1β can induce JNK signaling independently of NF-κB signaling. Inhibition of both IL-1 and TNF sensitizes the LSCs and leukemic progenitors to NF-κB inhibition. Furthermore, they showed that combined inhibition of TNF-α, IL-1, and NF-κB *in vivo* significantly impaired LSC function in the MLL-AF9 mouse model of AML, and prolonged survival in the secondary transplantation setting. Therefore, they suggested that inhibiting both TNF and IL-1β signaling could be a promising treatment for the M4/M5 subtypes of AML and for therapy-related AML (98).

However, the mechanism of TNF-α secretion and NF-κB activation in pre-LSCs and LSCs in MDS and progression to AML has been unclear. Gañán-Gómez et al. identified a cluster of microRNAs that regulate the expression of NF-κB in MDS (143). They detected significantly higher expression of miR-125a in MDS patients, and described a correlation between the expression of miR-125a and miR-99b, which is in the same cluster, with prognosis in MDS. They described the activation of NF-κB by miR-125a and miR-99b *in vitro*. However, the expression level of miR-99b and miR-125a showed a negative correlation with TLR2 and TLR7 RNA expression levels, which suggests that the activation of NF-κB by the miRNA clusters is independent of TLR signaling. In addition, they suggested that mir-125a inhibits NF-κB upon TLR stimulation, which could act as a fine-tuning mechanism for regulating NF-κB expression in MDS. Moreover, they demonstrated the inhibition of erythroid differentiation by miR-125a in MDS and in leukemia cell lines, which might make it a potential therapeutic target and prognostic marker for MDS (143).

## Clinical Applications of Modulating TNF-α/NF-κB Signaling

The potential of therapeutic targeting of NF-κB in AML has been explored in several early phase clinical trials (Table 2). One strategy used to inhibit NF-κB signaling is the proteasome inhibitor bortezomib, which is approved by the FDA for the treatment of multiple myeloma. Bortezomib inhibits NF-κB signaling by blocking the ubiquitin-proteasome pathway, which prevents degradation of phosphorylated IKB, the inhibitory protein of NF-κB. Stabilization of IKB prevents the nuclear translocation of NF-κB, which is necessary for its function as a transcription factor (144). Howard et al. reported the results of a phase I study with weekly bortezomib in combination with idarubicin in elderly patients with AML, most with preceding MDS. They demonstrated the feasibility of the combination of bortezomib and idarubicin and showed that this combination was well tolerated (80). Some clinical activity was observed, with a 20% complete remission rate, and with a decrease in circulating blasts in most patients. Their data suggest that inhibition of NF-κB and activation of p53 were associated with the activity of bortezomib and idarubicin in AML blasts. In another phase I study of bortezomib used in combination with induction chemotherapy in an older AML patient population, Attar et al. reported a CR rate of 61%, and a tolerable toxicity profile (79). However, in a phase II trial for pediatric relapsed refractory AML testing bortezomib in combination with chemotherapy, minimal clinical responses were observed (81). Additional clinical trials are underway testing bortezomib in combination with chemotherapy in pediatric AML. Therefore, the use of bortezomib needs to be further investigated, and may be effective in the correct clinical context. However, bortezomib is likely to have additional biological and clinical effects besides the inhibition of NF-κB signaling (144).

More specific inhibitors targeting NF-κB are currently in preclinical development. For example, the natural compound parthenolide has also been shown to inhibit NF-κB signaling, and was found to have preclinical activity in AML LSCs (139). Furthermore, Dai et al. showed that parthenolide enhances the lethality of pan-histone deacetylase inhibitors in AML, and that this strategy can target leukemic progenitor cells (145). In a recent study, Gao et al. determined that the small molecule CPPTL, a novel analog of parthenolide, causes cytotoxicity and apoptosis of AML cells *in vitro* (146). Furthermore, the CPPTL prodrug DMA-CPPTL prolonged survival in a patient-derived AML xenograft model. Their findings demonstrated that this drug induces the generation of ROS, followed by JNK pathway activation, which then promotes mitochondrial damage. These results suggest that CPPTL may be a promising drug candidate for the treatment of AML. The clinical testing of more specific inhibitors targeting TNF and NF-κB will shed more light on the clinical significance of this pathway in disease progression in myeloid malignancies.

## Concluding Remarks

There is now substantial evidence that a proinflammatory microenvironment, which can be initiated through cytokine and chemokine secretion both by hematopoietic cells and by stromal cells, can promote the emergence of pre-LSCs and progression to AML. While intriguing, further studies are needed to make a clear connection between inflammation and the pathogenesis or minimally known clinical manifestations of clonal hematopoiesis. It is unclear why the majority of patients with clonal hematopoiesis do not progress despite having mutations that are common in MDS and AML. It is possible a “second hit” is needed to generate pre-LSCs or LSCs, and that cells with a single mutation have increased self-renewal properties but limited clonal expansion capacity (147). Furthermore, studying clonal progression and pre-LSCs in aseptic mouse models can be difficult, as these models do not accurately represent the more infection-prone environment in which most humans normally reside. The low-grade chronic inflammatory state of aging may contribute both to the initiation of preleukemic mutations, as well as to the clonal outgrowth of mutated cells, which in some cases can progress to the preleukemic or leukemic state. Current efforts are underway to generate better murine models of clonal and preleukemic hematopoiesis to improve our understanding of the roles of inflammatory signaling pathways in leukemic progression.

Preleukemic or leukemic cells can then propagate the disease by interacting with the microenvironment, leading to the increased secretion of proinflammatory factors, such as S100A8/S100A9, IL-1β, IL-6, and IL-8. Furthermore, while inflammatory signaling is a critical component of the crosstalk between HSPCs and their microenvironment during infection and hematopoietic stress, chronic activation of inflammatory signaling pathways such as IFN-α, IFN-γ, TLR, and TNF-α can also suppress normal HSC function and lead to bone marrow failure. In the case of IFN-α/β, the ability to drive HSPCs into the cell cycle similarly affects LSCs, and can be exploited to impair their self-renewal to aid in their elimination, leading to long-term remissions in MPNs and other myeloid malignancies. Many clinical trials are now testing this strategy for relapse prevention, in some cases in combination with other therapeutic agents, with some early promising results. Currently, combinations strategies that include IFN-α/β appear to be the most promising immunomodulating approaches for the treatment of hematologic malignancies, such as those combining JAK2 inhibitors with IFN-α/β for MPN, with a real possibility of inducing long-term remissions. The experience with the JAK2 inhibitor ruxolitinib as a single agent in MPN suggests that inhibiting a single inflammatory signaling pathway may not be sufficient to achieve long-term results (9). Similar combination strategies including antiproliferative therapies together with immunomodulating agents need to be more widely explored both preclinically and in the clinic.

There is convincing preclinical data that dampening of some inflammatory signaling pathways, such as IL-1β, IL-6, and IL-8, can eliminate LSCs and in some cases induce differentiation of AML blasts. While inhibitory antibodies or pharmacological inhibitors are available to block these pathways (see Figure 1 and Table 2), their clinical translation has been challenging, due to the context-dependent effects of some of these inflammatory signals. For example, ILβ is likely to have dose-dependent effects on the proliferation and differentiation of hematopoietic cells, and appears to have differential effects on normal HSPCs, pre-LSCs in MPN, and LSCs and leukemic blasts in AML. On the other hand, other inflammatory signals, particularly TLRs, can also become downregulated with progression to AML, and re-expression of these factors in leukemic cells can lead to differentiation. In this case, TLR antagonists may be effective in preventing the progression of early MDS, while TLR agonists may be more useful for promoting the differentiation of AML blasts. Therefore, each potential therapeutic agent modulating one of these inflammatory signaling pathways needs to be tested in the correct disease stage and context, and careful attention must be paid to dosing and timing of treatment, since levels of pathway activation may have distinct effects on the proliferation and differentiation of pre-LSC and LSCs.

## Author Contributions

All authors contributed to the conception, writing, and editing of the manuscript, and have approved it for publication.

## Conflict of Interest Statement

The authors declare that the research was conducted in the absence of any commercial or financial relationships that could be construed as a potential conflict of interest. The handling editor declared a shared affiliation, though no other collaboration, with the authors.
